# Ammonium chloride alters neuronal excitability and synaptic vesicle release

**DOI:** 10.1038/s41598-017-05338-5

**Published:** 2017-07-11

**Authors:** Roman M. Lazarenko, Claire E. DelBove, Claire E. Strothman, Qi Zhang

**Affiliations:** Department of Pharmacology, Vanderbilt University, 23rd Avenue South at Pierce Street, Nashville, TN 37232 USA

## Abstract

Genetically encoded pH-sensors are widely used in studying cell membrane trafficking and membrane protein turnover because they render exo-/endocytosis-associated pH changes to fluorescent signals. For imaging and analysis purposes, high concentration ammonium chloride is routinely used to alkalize intracellular membrane compartments under the assumption that it does not cause long-term effects on cellular processes being studied like neurotransmission. However, pathological studies about hyperammonemia have shown that ammonium is toxic to brain cells especially astrocytes and neurons. Here, we focus on ammonium’s physiological impacts on neurons including membrane potential, cytosolic Ca^2+^ and synaptic vesicles. We have found that extracellularly applied ammonium chloride as low as 5 mM causes intracellular Ca^2+^-increase and a reduction of vesicle release even after washout. The often-used 50 mM ammonium chloride causes more extensive and persistent changes, including membrane depolarization, prolonged elevation of intracellular Ca^2+^ and diminution of releasable synaptic vesicles. Our findings not only help to bridge the discrepancies in previous studies about synaptic vesicle release using those pH-sensors or other vesicle specific reporters, but also suggest an intriguing relationship between intracellular pH and neurotransmission.

## Introduction

The proton (H^+^) gradient provides the driving force for many cellular processes. For example, the lumen of synaptic vesicles (SVs) responsible for neurotransmitter release have a very low pH (~5.5) essential for neurotransmitter import^1^. The pH difference between intracellular organelles and the extracellular environment has been harnessed for the study of membrane and protein turnover. The invention of pHluorin, the first genetically encoded pH sensor, greatly facilitated imaging-based approaches for such studies^2^. Since then, pH-sensitive fluorescence proteins (pH-FPs), inserted into the extracellular/luminal domain of selected membrane proteins, have been extensively used to monitor the turnover of SVs, secretory vesicles and endosomes as well as the membrane proteins themselves *in vitro* and *in vivo* ^3–5^. For optimal pH-sensitivity within the physiological range (pH 5.5–8), most pH-FPs have a pK_a_ (i.e., the pH value at which the fluorescence is 50% of maximal) around 7 and are nearly non-fluorescent at pH 5.5 (the pH of most secretory vesicles and recycling endosomes)^6, 7^. Hence, it is necessary to artificially neutralize all intracellular membrane compartments in order to visualize and quantify all pH-FPs expressed. For that, high concentration (e.g. 50 mM) ammonium chloride (NH_4_Cl) is routinely used. NH_4_^+^ is a weak base (pK_a_ = 9.24) and readily dissociates into H^+^ and cell-permeable ammonia gas (NH_3_) in neutral pH. After diffusing into acidic organelles, NH_3_ quickly turns back to NH_4_^+^ by sequestering intracellular H^+^, causing deacidification^8^. Removing extracellular NH_4_^+^ results in the exit of NH_3_ from the intracellular compartments, leaving H^+^ behind (i.e. reacidification). Since the conversion between NH_4_^+^ and NH_3_ is instantaneous and reversible, it is generally assumed that the cellular impacts of NH_4_Cl, if any, are transient and reversible. As such, high concentration NH_4_Cl is also applied prior to fluorescence imaging for selecting imaging fields and adjusting acquisition settings (e.g. focus and exposure time)^9^. This practice is particularly necessary for pH-FP-based study of SVs in neurons^2, 10–22^ because (1) many presynaptic terminals reside along long and complex neuronal axons, (2) SVs and presynaptic terminals are tiny (~40 nm and 1 μm respectively) and (3) pH-FPs tagged to the luminal domains of SV-specific proteins like Synaptophysin are almost completely quenched and thus non-fluorescent at pH 5.5^23–26^. For quantitative measurement, repeated applications of high concentration NH_4_Cl are also necessary in studying trafficking and cell surface distribution of membrane proteins including receptors, ion channels and transporters over time^3^.

While extracellular application of NH_4_Cl is a convenient and popular method to manipulate intracellular pH, the assumption that ammonia or ammonium has little effect on neurons or its effect is transient and reversible remains untested. In fact, there are reasons to believe that NH_4_Cl can profoundly alter neurotransmission. First, the substitution of Na^+^ by NH_4_^+^ (for making high-concentration NH_4_Cl solutions) may affect membrane potential (E_m_) through Na^+^-sensitive leak channels like NALCN as well as a variety of other ion channels. Second, hydrated NH_4_^+^ with an ionic radius identical to K^+^ (1.45 Å)^27, 28^ may compete with K^+^ for binding to K^+^-channels and transporters^29^. Third, NH_3_ can activate Na^+^-K^+^-2Cl^−^ cotransporter isoform 1 (NKCC1) and impair K^+^ buffering^30^. Fourth, NH_4_Cl-induced pH changes in mitochondria may disrupt intracellular Ca^2+^ homeostasis and respiratory bioenergetics^31^. Pathologically, a millimolar increase of extracellular ammonia is known to cause over-excitation and disinhibition of neural circuitry, leading to neurotoxicity in hyperammonemic encephalopathy^30, 32^. Hence, it is necessary to address if NH_4_Cl has a long-lasting or irreversible impact on neurotransmission since it is so widely used in conjunction with pH-FPs in studying SVs and many other aspects of neurotransmission. Here, we measured the effects of three concentrations (5, 10 and 50 mM) of NH_4_Cl on neuronal E_m_, intracellular Ca^2+^ concentration ([Ca^2+^]_i_) and, most importantly, SV release using whole-cell patch clamp recording and live-cell fluorescence imaging. We have found that 50 mM NH_4_Cl induces significant membrane depolarization, [Ca^2+^]_i_ increase and SV release. After thorough washout, increases in [Ca^2+^]_i_ and exhaustion of releasable SVs persists. At the lowest concentration tested (5 mM), NH_4_Cl induces a significant increase of [Ca^2+^]_i_ and reduces SV release long after the washout. Together, our results demonstrate that NH_4_Cl has profound and long-lasting effects on [Ca^2+^]_i_ and presynaptic SV release, which complicates SV turnover and synaptic transmission.

## Results

**Figure 1:**
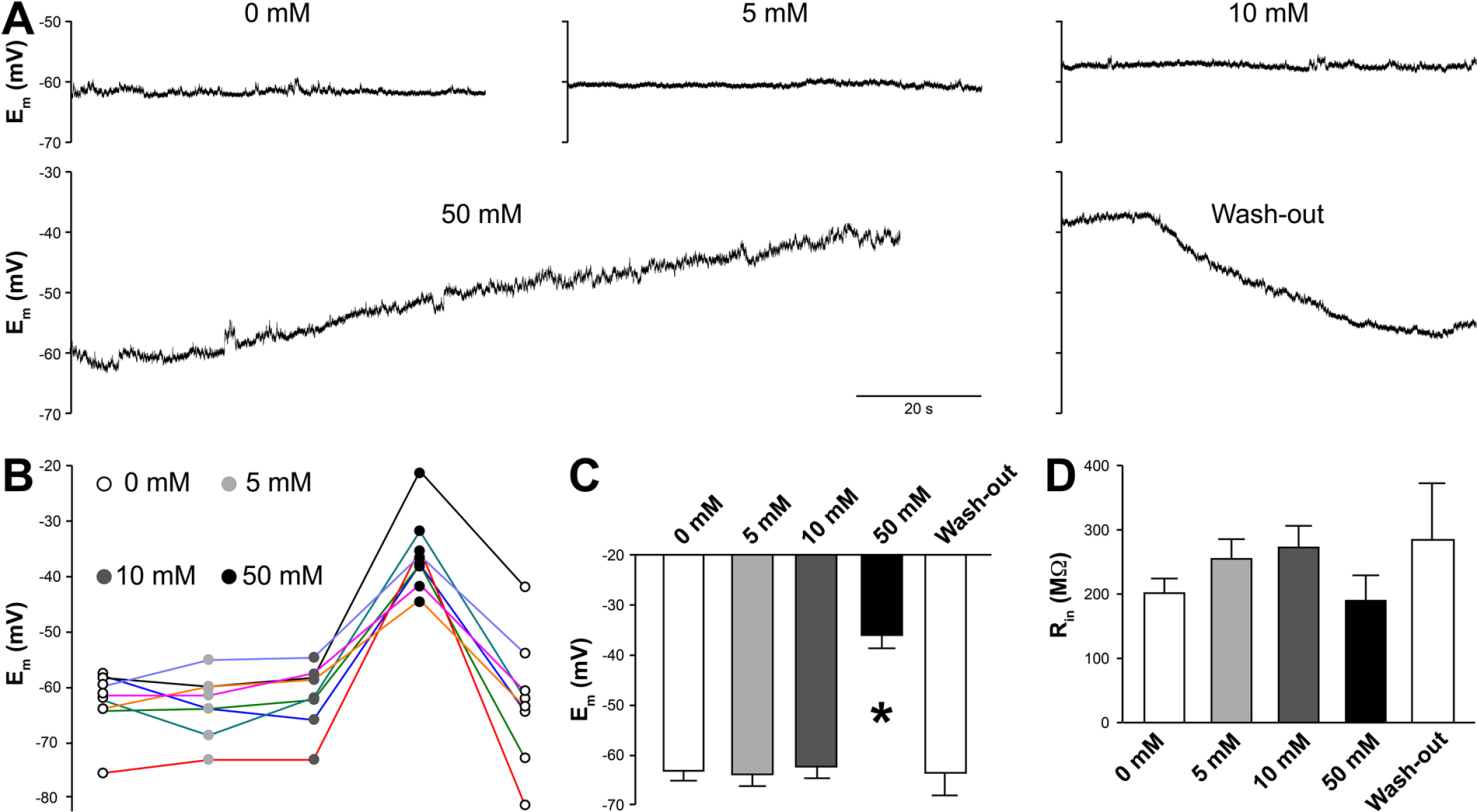
Effects of ammonium chloride (NH_4_Cl) on passive membrane properties. (**A**) Sample traces of resting membrane potential (E_m_) recorded continuously in current-clamped neurons in the presence of synaptic blockers and TTX with increasing concentrations of NH_4_Cl (5–10–50 mM). (**B**) Changes of E_m_ in neurons exposed to NH_4_Cl. (**C**) Averaged E_m_s in response to NH_4_Cl, all of which were measured at 5 minutes after the applications. Only 50 mM caused slow and reversible depolarization (to ~−35 mV, n = 8 neurons, *p* < 0.01, *F* = 20.28, one-way ANOVA). (**D**) None of the NH_4_Cl treatments changed the input resistance (R_in_) of the recorded neurons (n = 8 neurons, *p* = 0.43, *F* = 0.98, one-way ANOVA).

Using rat hippocampal cultures, we first surveyed neuronal E_m_ in the presence of different concentrations (5, 10 and 50 mM) of extracellularly applied NH_4_Cl. Following the procedure reported in the literature^2, 10–21^, we prepared NH_4_Cl solutions by substituting an equal amount of NaCl with NH_4_Cl. To obtain an uninterrupted readout of E_m_, we used tetrodotoxin (1 μM), bicuculline (10 μM), NBQX (10 μM) and D-AP5 (20 μM) to block action potentials as well as inhibitory and excitatory postsynaptic currents. Normal Tyrode’s solution and three different concentrations of NH_4_Cl (5, 10 and 50 mM) were applied sequentially during recording. 5 and 10 mM NH_4_Cl induced little or no change to E_m_ (−63.2 ± 2.1 and −61.5 ± 2.2 mV *vs* −63 ± 2.1 mV in normal Tyrode’s solution, n = 8 neurons). However, 50 mM NH_4_Cl consistently led to gradual but substantial depolarization (to −35.7 ± 2.7 mV, n = 8 neurons) which slowly recovered in the subsequent washout with normal Tyrode’s solution (Fig. 1A–C). On average, it took 446.1 ± 45.4 seconds for E_m_ to reach a steady state of depolarization; during washout, it took 223.3 ± 41.0 seconds on average for E_m_ to recover. The NH_4_^+^ blockade of barium-sensitive potassium channels^29^ or competition for K^+^ channels and transporters due to its size similarity^27, 28, 33^ may have contributed to the relatively slow depolarization and repolarization. In addition, the substitution of Na^+^ by NH_4_^+^ may influence Na^+^-sensitive background sodium leak channel NALCN^34^, leading to membrane potential fluctuation. At the end of each NH_4_Cl treatment for every neuron recorded, we also measured input resistance (R_in_) to check cell membrane integrity. We did not observe a statistically significant difference in R_in_ among the four conditions (Fig. 1D), suggesting that NH_4_Cl does not break the plasma membrane or significantly change its ion conductance. Switching between normal Tyrode’s solution and the three different NH_4_Cl solutions in the absence of the cocktail of synaptic blockers often changed neuronal firing pattern, which is likely determined by the combination of excitatory and inhibitory inputs each neuron received (data not shown). Together, the electrophysiological data showed a clear effect of 50 mM NH_4_Cl on neuronal E_m_ and thus prompted us to examine [Ca^2+^]_i_ and SV release in neurons exposed to NH_4_Cl.

**Figure 2:**
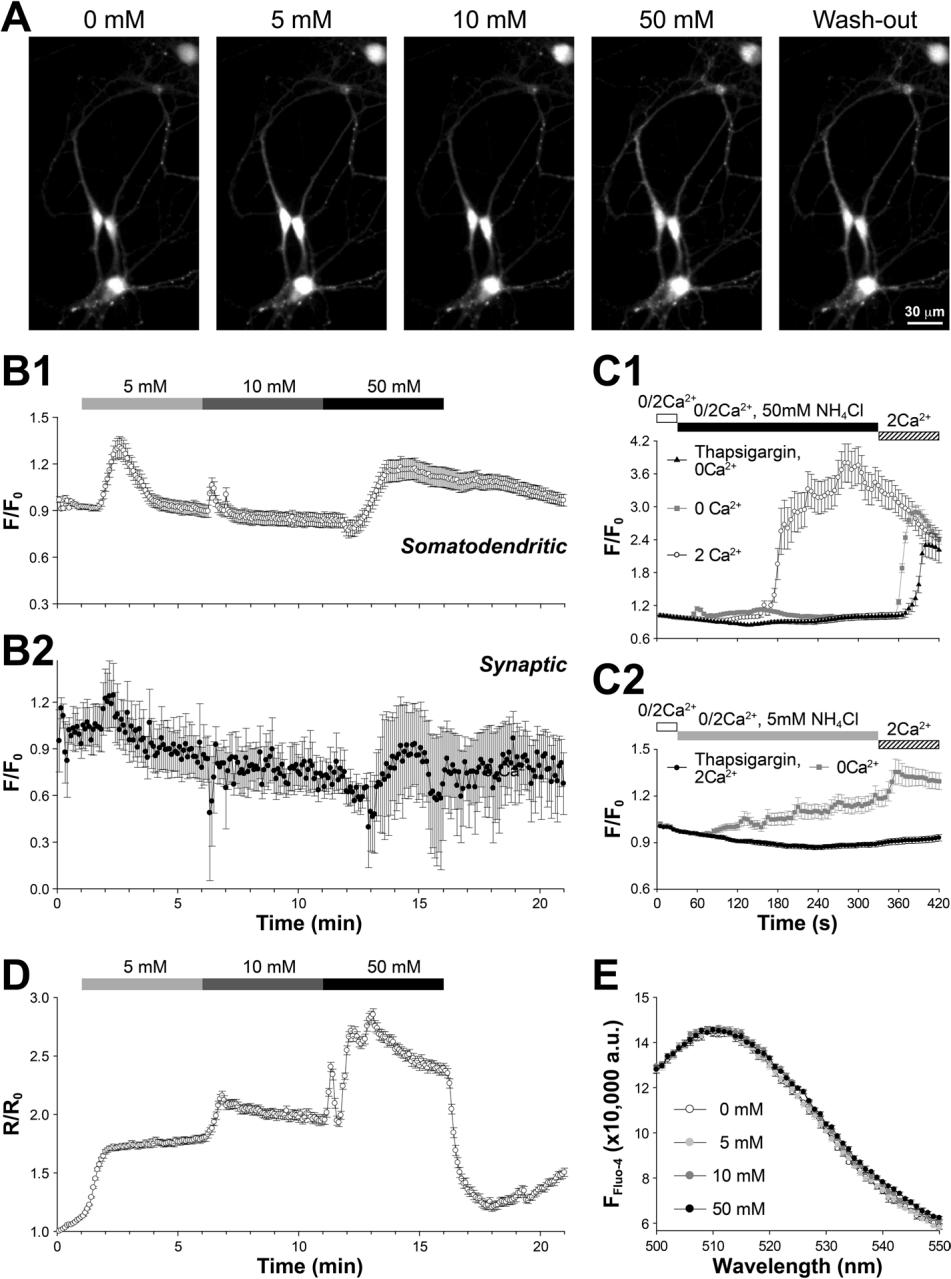
Effects of NH_4_Cl on intracellular calcium ([Ca^2+^]_i_). (**A**) Sample images on neurons preloaded with Fluo-4-AM and sequentially exposed to different concentrations of NH_4_Cl. (**B**) Fluo-4 fluorescence change (normalized to the pretreatment baseline) in response to different concentrations of NH_4_Cl as indicated (n = 300 randomly selected synaptic boutons from 6 independent trials). (**B1**) In somatodendritic regions 5 mM NH_4_Cl caused strong (~34.2%) but transient increase of Fluo-4 fluorescence. The response to subsequent 10 mM NH_4_Cl application was smaller, whereas 50 mM NH_4_Cl lead to a [Ca^2+^]_i_ increase comparable to that of 5 mM NH_4_Cl but lasted even after the 5-min wash-off. (**B2**) [Ca^2+^]_i_ change in SypHTm-defined synaptic areas resembled that of somatodendritic areas. In both cases, a gradual decrease of Fluo-4 fluorescence occurred likely due to photobleaching and dye loss. (**C**) NH_4_Cl-induced [Ca^2+^]_i_ changes during 0 Ca^2+^ or after thapsigargin pretreatment. (**C1**) The prolonged [Ca^2+^]_i_ increase (white circles) caused by 50 mM NH_4_Cl was diminished with 0 Ca^2+^ (gray squares), and the resupply of 2 mM extracellular Ca^2+^ led to a large [Ca^2+^]_i_ increase with or without thapsigargin pretreatment (black triangles). For each group, 10 ROIs/FOV were randomly selected and 4 FOVs were analyzed. (**C2**) The transient [Ca^2+^]_i_ increase caused by 5 mM NH_4_Cl was significantly reduced with 0 Ca^2+^ (gray squares), and the resupply of 2 mM extracellular Ca^2+^ led to additional [Ca^2+^]_i_ increase. Thapsigargin pretreatment eliminated those two [Ca^2+^]_i_ increases (black circles). For each group, 10 ROIs/FOV were randomly selected and 4 FOVs were analyzed. (**D**) Cytosolic pH changes upon various concentrations of NH4Cl were measured by ratiometric imaging of BCECF fluorescence (R = F_480_/F_440_, i.e. excited at 440 and 480 nm, and fluorescence imaged with 510 ± 10 nm band-pass emission filter), which was further normalized to the initial R_0_ before any treatments. The cytosolic pH changes are very different from [Ca^2+^]_i_ change reported by Fluo-4 (**B**). (**E**) Spectrofluorometry scan of F_Fluo__-4_ emission with 480 nm excitation in the presence of different concentrations of NH_4_Cl as indicated. Four repeats for every concentration.

To study [Ca^2+^]_i_ changes, we preloaded hippocampal cultures with Fluo-4-AM, a membrane-permeable green fluorescence Ca^2+^ indicator. The same sequential NH_4_Cl application used in the electrophysiological experiments was performed. First, we focused on somatodendritic areas where basal Fluo-4 fluorescence could be readily distinguished from background. Surprisingly, 5 mM NH_4_Cl caused a transient but significant [Ca^2+^]_i_ increase (~34% above the pretreatment baseline) at somatodendritic areas, whereas subsequent 10 mM NH_4_Cl induced a much smaller [Ca^2+^]_i_ spike. The reduction of [Ca^2+^]_i_ increase in 10 mM NH_4_Cl is expected if the source of Ca^2+^ is internal Ca^2+^ stores that could be exhausted in the end of 5 mM NH_4_Cl application or if there were an increase of the plasma membrane Ca^2+^ conductance that could be suppressed in the end of 5 mM NH_4_Cl. The final 50 mM NH_4_Cl consistently induced a delayed but significant [Ca^2+^]_i_ elevation with amplitudes comparable to that of 5 mM NH_4_Cl. Importantly, this [Ca^2+^]_i_ elevation persisted even during the subsequent 5-minute washout (Fig. 2A and B1 and Supplementary Video 1), resembling the slow E_m_ depolarization and repolarization previously observed. Using the synaptic marker Synaptophysin-pHTomato (SypHTm, described below), we also identified the synaptic boutons and found that synaptic Fluo-4 exhibited the same fluorescence change as those seen in somatodendritic regions (Fig. 2B2), indicating a similar impact of NH_4_Cl on synaptic [Ca^2+^]_i_. Notably, it has a larger variation, likely due to much smaller synaptic volume and thus much lower fluorescence signal.

To probe the source of Ca^2+^, we applied 50 mM NH_4_Cl with or without extracellular Ca^2+^. With normal Tyrode’s solution, the resulting [Ca^2+^]_i_ increase was again delayed but much larger (Fig. 2C1, white circles), suggesting an inhibitory or desensitizing effect of the preceding 5 and 10 mM NH_4_Cl in the sequential application. 0 Ca^2+^ Tyrode’s solution eliminated the large and delayed response (Fig. 2C1, gray squares), indicating that 50 mM NH_4_Cl-induced [Ca^2+^]_i_ increase is mostly due to the slow E_m_ depolarization and Ca^2+^ influx through voltage-gated Ca^2+^ channels (VGCCs). Interestingly, there was a small but quick [Ca^2+^]_i_ increase in 0 Ca^2+^ bath solution, implicating a small contribution of Ca^2+^ from the internal stores especially in the absence of extracellular Ca^2+^. Notably, perfusion of normal Tyrode’s solution after 0 Ca^2+^ and 50 mM NH_4_Cl caused an immediate and large [Ca^2+^]_i_ increase, consistent with the notion that VGCCs remain open as the recovery of E_m_ was very slow. Furthermore, we pretreated cells with 1 μM thapsigargin to deplete the internal Ca^2+^ stores. Expectedly, the [Ca^2+^]_i_-response to 50 mM NH_4_Cl was completely abolished by 0 Ca^2+^ and thapsigargin, and the response to the normal Tyrode’s washout was also reduced and delayed (Fig. 2C1, black triangles), implying an inhibition of VGCCs by internal store depletion^35^.

Next, we probed the source of [Ca^2+^]_i_ increase by low or mild NH_4_Cl. While extracellular Ca^2+^ was removed before and during 5 mM NH_4_Cl application, there was a smaller and gradual [Ca^2+^]_i_ increase, and the subsequent normal Tyrode’s washout caused an additional small increase of [Ca^2+^]_i_ (Fig. 2C2, gray squares), which seemed to be faster than a compensatory [Ca^2+^]_i_ increase in transition from 0 Ca^2+^ to normal Tyrode’s without NH_4_Cl (Fig. S1). However, 1-μM thapsigargin pretreatment resulted in a complete loss of [Ca^2+^]_i_ increase by 5 mM NH_4_Cl even in the normal Tyrode’s (Fig. 2C2, black circles). These results suggest that the 5 mM NH_4_Cl-induced [Ca^2+^]_i_ increase is also a result of both Ca^2+^ release from internal stores and cell membrane Ca^2+^-conductance change, which is transient and likely regulated by internal Ca^2+^ stores. Further investigation is certainly needed on these complicate [Ca^2+^]_i_ effects.

We also examined the possibility that the changes of Fluo-4 signals were caused by intracellular pH or NH_4_^+^ changes instead of [Ca^2+^]_i_ fluctuations. Generally, ammonium converts to ammonia instantaneously, readily crosses the plasma membrane and causes an immediate intracellular pH change. Furthermore, the volume of bath solution vastly exceeds that of the cytoplasm, meaning that the supply of ammonia overrides any intracellular buffering power. Therefore, if the Fluo-4 fluorescence change had been caused by intracellular pH change, it should have increased instantaneously, stayed elevated during the NH_4_Cl treatments and fallen back immediately upon washout. We directly monitored the cytosolic pH using BCECF-AM (2′,7′-Bis-(2-Carboxyethyl)-5-(and-6)-Carboxyfluorescein, Acetoxymethyl Ester), a membrane-permeable and ratiometric pH indicator ideal for measuring intracellular pH^36^. Cells preloaded with BCECF-AM were subjected to the sequential NH_4_Cl treatments as previously described, and cellular BCECF fluorescence under 440 nm (isosbestic point) and 480 nm excitations were obtained to calculate F_480_/F_440_ (Fig. S2), which reports intracellular pH change^37^. As expected, the increase and decrease of F_480_/F_440_ were synchronized with the application and washout of NH_4_Cl (Fig. 2D) and very different from Fluo-4 fluorescence change. In addition, spectrofluorometry measurement showed that Fluo-4 fluorescence was insensitive to NH_4_Cl (Fig. 2E), excluding direct interference by NH_4_^+^. Therefore, we conclude that the Fluo-4 signals indeed represent NH_4_Cl-induced [Ca^2+^]_i_ changes.

**Figure 3:**
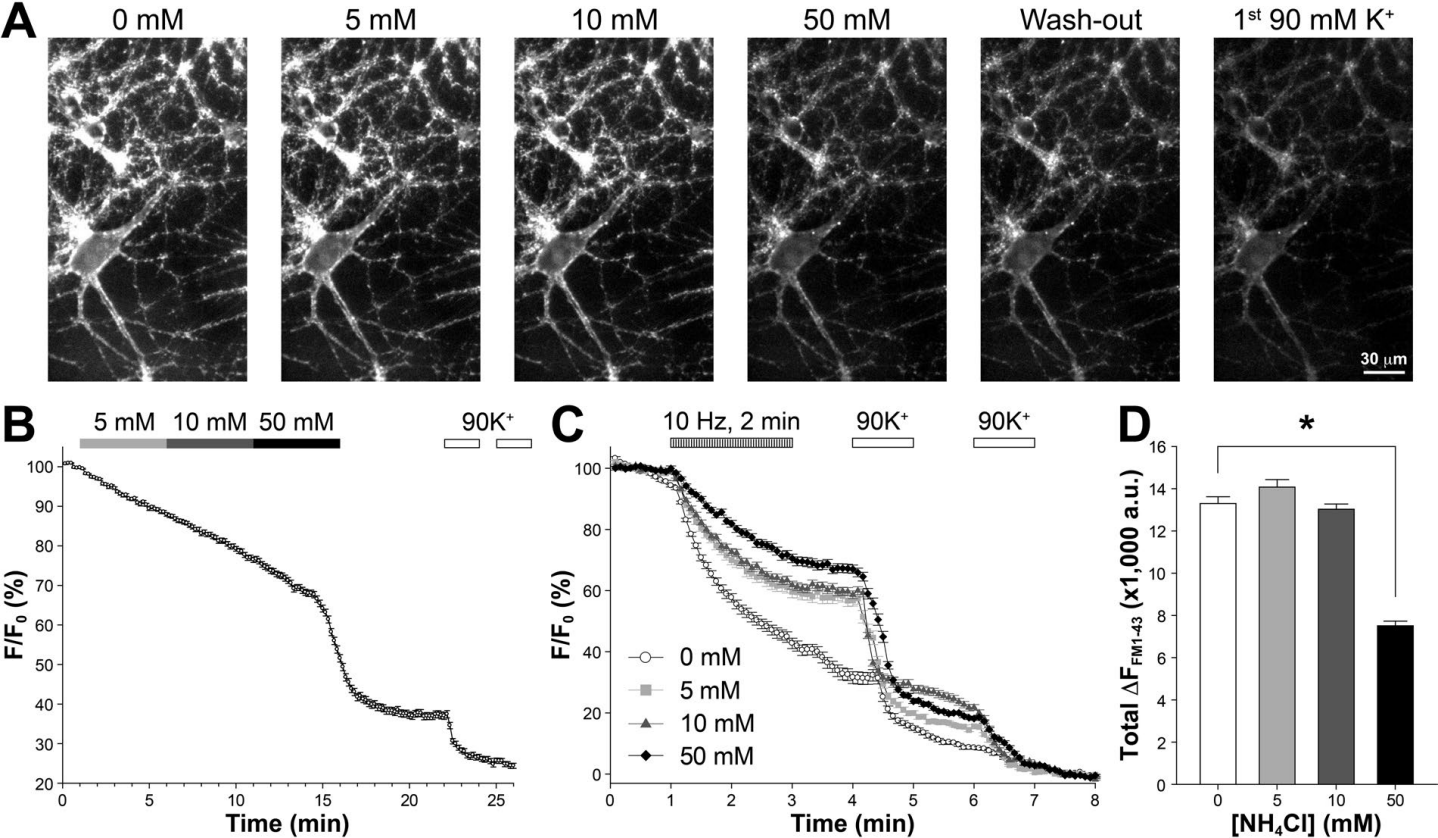
Effects of NH_4_Cl on synaptic vesicle (SV) release. (**A**) Sample images of neurons preloaded with FM1-43 dye and exposed to NH_4_Cl-containing bath solutions and 90 mM K^+^ modified Tyrode’s saline. (**B**) Averaged time-course of FM1-43 fluorescence (normalized to pretreatment baseline) during the perfusion of increasing concentrations of NH_4_Cl and 90 mM K^+^ (randomly selected 90 ROIs/FOV and 6 FOVs). There was a gradual decay of FM1-43 fluorescence in the presence of three concentrations of NH_4_Cl, but only in the presence of 50 mM NH_4_Cl did it show a delayed but significant decrease of FM1-43 fluorescence (~22% in contrast to 7% in 5 and 10 mM NH_4_Cl, which was likely attributed to photo-bleaching of the fluorophore) and further reductions in 90 mM K^+^ (~32%). (**C**) After 5-min incubation with various concentrations of NH_4_Cl and 5-min washout with normal Tyrode’s solution, FM1-43 was destained by sequential stimuli (10-Hz 2-min field electrical stimulation and two consecutive 1-min 90 mM K^+^) in the indicated order (for each group, randomly selected 79 ROIs/FOV and 3 FOVs). (**D**) Averaged total FM1-43 fluorescence loss in those synaptic boutons after all three stimuli (10-Hz 2-min field electrical stimulation and two consecutive 1-min 90 mM K^+^). 50 mM NH_4_Cl significantly reduced the total FM1-43 destaining (*p* < 0.05, Student’s *t*-test).

The fact that NH_4_Cl causes E_m_ and [Ca^2+^]_i_ changes prompted us to examine its impact on SV release and retrieval. First, we loaded SVs with FM1-43 (a styryl dye commonly used to study SV release)^38^ and continuously monitored its loss during the sequential NH_4_Cl application as before. We did not detect a typical exponential decay of FM1-43 fluorescence during 5 and 10 mM NH_4_Cl treatments, consistent with the idea that such concentrations of extracellular NH_4_Cl are insufficient to activate VGCCs and to cause evoked SV release. Instead, there was a progressive dye loss (about 7% under 5 or 10 mM NH_4_Cl) faster than the photobleaching seen in the pretreatment baseline (Fig. 3A and B and Supplementary Video 2). This can be explained by (1) an increase of spontaneous SV release due to baseline [Ca^2+^]_i_ increase^39–44^, (2) the slow propagation of [Ca^2+^]_i_ increase from somatodendritic areas to distal axons, and (3) the high affinity Synaptotagmins regulating spontaneous release^45, 46^. Further investigation is needed to appraise the effects of low or moderate concentrations of NH_4_Cl in spontaneous SV release. With 50 mM NH_4_Cl, we observed a delayed but substantial (~34%) FM1-43 destaining with an exponential decay resembling that of evoked SV release (Fig. 3A and B and Supplementary Video 2). Again, the delay is temporally matched to slow E_m_ depolarization by 50 mM NH_4_Cl. The significant FM1-43 loss again persisted during the subsequent washout with normal Tyrode’s solution containing NBQX and D-AP5, likely because neuronal E_m_ recovered rather slowly. Final FM1-43 loss (~30%, also exponential) due to 90 mM K^+^ (with 10 μM NBQX and 20 μM D-AP5) (Fig. 3A and B and Supplementary Video 2) is similar to the 50 mM NH_4_Cl-induced loss, suggesting that 50 mM NH_4_Cl indeed evoked the release of SVs belonging to the releasable pool^47^.

The long-lasting impacts of NH_4_Cl motivated us to ask if and how a prior NH_4_Cl treatment followed by a complete washout would change evoked SV release. Following FM1-43 loading (90 mM K^+^, 2 minutes) and surface dye washout, we treated cells with 0, 5, 10 and 50 mM NH_4_Cl (containing NBQX and D-AP5) for 5 minutes and applied another 5-minute washout with dye-free normal Tyrode’s solution (also containing NBQX and D-AP5), simulating a common scenario in which NH_4_Cl pretreatment is used to bring up pH-FP fluorescence before imaging. We monitored FM1-43 fluorescence while 2-minute 10-Hz electric field stimulation and two 1-minute 90 mM K^+^ (containing NBQX and D-AP5) were applied sequentially. We found that FM1-43 destaining was inversely correlated to the concentration of NH_4_Cl (Fig. 3C), suggesting that higher concentration of NH_4_Cl lowers the SV release probability even after 5-minute recovery. 90 mM K^+^ treatments, causing maximal exocytosis of the total releasable pool of SVs, led to a total loss of remaining FM1-43 (Fig. 3C). We also calculated the total FM1-43 fluorescence loss (total ΔF_FM1-43_, corresponding to all dye-labeled SVs that underwent evoked release) by subtracting the final residual FM1-43 fluorescence from the fluorescence before the electrical field stimulation. Only 50 mM NH_4_Cl pretreatment caused a significant reduction of total ΔF_FM1-43_ (Fig. 3D), consistent with the previous observation that only 50 mM NH_4_Cl induced evoked release of releasable SVs (Fig. 3B). To be noted, the similarity of total ΔF_FM1-43_ among 0, 5 and 10 mM is not in conflict with the previously observed progressive FM1-43 loss (Fig. 3B) because the total ΔF_FM1-43_ represents SVs that underwent evoked release and is likely different from SVs that release spontaneously^42^. Together, the FM1-43 results suggest that the effect of NH_4_Cl in all three concentrations can cause prolonged interference with SV release, even after 5-min washout/recovery.

**Figure 4:**
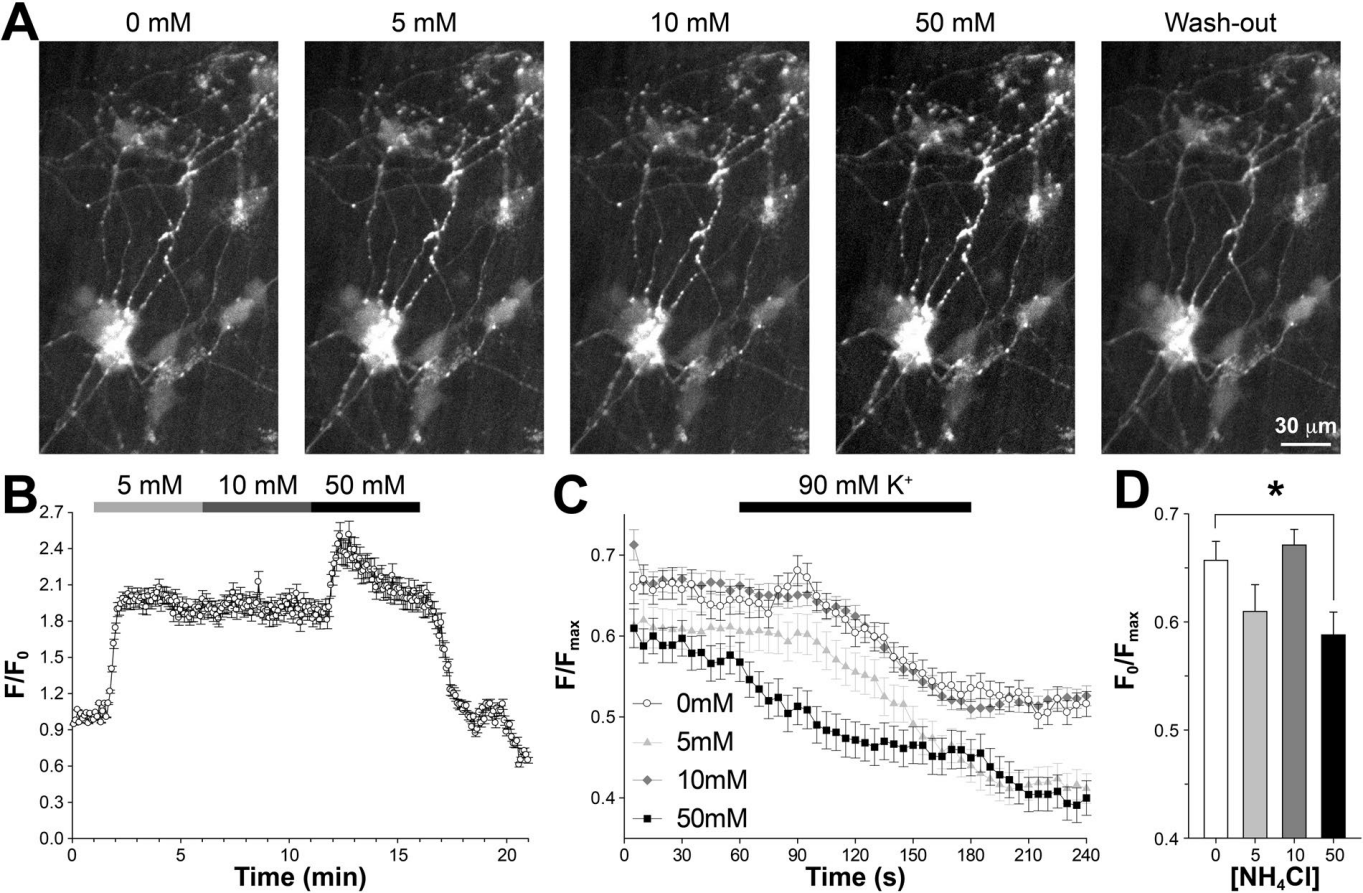
Effects of NH_4_Cl on SypHTm – labeled SVs. (**A**) Sample images of neurons transfected with SypHTm construct and exposed to various concentrations of NH_4_Cl. (**B**) Averaged time-course of the SypHTm fluorescence change (n = 300 randomly selected synaptic boutons from 6 independent trials), reflecting the de-acidification that SypHTm experiences. There was a substantial and sustained increase of fluorescence in 5 mM NH_4_Cl (~100%) but little change upon 10 mM NH_4_Cl, and a further increase (~60%) upon 50 mM NH_4_Cl application. Final wash-off caused two-phase fluorescence decrease below the initial baseline. (**C**) The relative change of SypHTm fluorescence in response to 90 mM K^+^ stimulation after cells’ pretreatment with 0, 5, 10 and 50 mM NH_4_Cl for 5 minutes and washout for another 5 minutes. SypHTm fluorescence increase only occurs in 0 mM group (for each group, randomly selected 35 ROIs/FOV and 3 FOVs). For every ROI, F_max_ (used as 1.0 for normalization) is defined as the fluorescence intensity difference between 50 mM NH_4_Cl and pH 5.5 Tyrode’s solution, both of which were applied sequentially at the end of imaging. (**D**) For those ROIs, the average SypHTm fluorescence before 90 mM K^+^ stimulation (F_0_) is normalized to F_max_. Only the 50 mM NH_4_Cl pretreatment caused a significant decrease (*p* < 0.05, ANOVA and Student’s *t*-test).

Last, we studied how NH_4_Cl would change SV release measured by pH-FPs. We used Synaptophysin (a SV specific protein) with luminally tagged pHTomato (SypHTm). The reason to choose pHTomato instead of pHluorin is that the former has a pK_a_ about 7.8 and thus remains partially fluorescence at 5.5 pH^48^, allowing us to locate SypHTm-positive synaptic boutons in expressing neurons and adjust imaging settings without needing NH_4_Cl pretreatment (Fig. 3A). Notably, SypHTm exhibits a smaller pH-sensitivity than most pH-FPs due to its high pK_a_^48^. The application of 5 mM NH_4_Cl immediately caused a significant and sustained increase (~100%) of SypHTm fluorescence (Fig. 4A and B and Supplementary Video 3), in good agreement with an NH_4_Cl-induced persistent SV deacidification. Little increase of SypHTm fluorescence was observed with the subsequent application of 10 mM NH_4_Cl (Fig. 4 and Supplementary Video 3), suggesting that 10 mM NH_4_Cl did not deacidify the SV lumen any further. However, the final application of 50 mM NH_4_Cl led to a further fluorescence increase of ~60%, which rose quickly but also fell partially during the whole 5-minute application (Fig. 4B and Supplementary Video 3). This suggests that neither 5 nor 10 mM NH_4_Cl de-acidifies the SV lumen completely and are inadequate to maximize pH-FPs fluorescence. Interestingly, the final washout lead to a two-phase fluorescence decrease (Fig. 4B). The first phase likely reflects the fast re-acidification upon NH_4_Cl withdrawal, and the second went well below the pretreatment baseline, implying a delayed compensatory endocytosis after SV release^49^.

To further understand how NH_4_Cl pretreatment affects SV behavior, we did the same NH_4_Cl pretreatments with 5-minute washout as previously described. During imaging, we applied 2-minute 90 mM K^+^ as stimulation. We observed a small but significant pHTm fluorescence increase (Fig. 4C, open circle) as expected (for the reason of partial pH-quenching described previously) in control (i.e. 0 mM NH_4_Cl/normal Tyrode’s solution). To normalize pHTm fluorescence across synaptic boutons, we used 50 mM NH_4_Cl and pH 5.5 Tyrode’s solution at the end to obtain total pHTm and baseline fluorescence respectively. The difference between the two represents pHTm inside acidic membrane-bound organelles like SVs (i.e. F_max_, set as 1.0 for normalization). We observed swollen synaptic boutons in 50 mM NH_4_Cl treated samples. Since any synaptic boutons that did not respond to the final 50 mM NH_4_Cl were likely unhealthy and could not maintain vesicular pH gradient, we eliminated those synaptic boutons in our analyses. Intriguingly, we observed pHTm fluorescence decrease instead of increase during 90 mM K^+^ stimulation in all three NH_4_Cl pretreatments and the decreases seemingly correlated to the concentrations (Fig. 4C). Moreover, 50 mM NH_4_Cl caused a significant decrease of surface fraction of pHTm before stimulation (Fig. 4D), suggesting a trapping of SypHTm in non-releasable membrane compartments like endosomes. While these results are seemingly different from the FM1-43 data, two factors should be considered: (1) pHTm exhibits much smaller pH-sensitivity than pHluorins and thus it is more difficult to detect small amount of SVs being released; and (2) FM1-43 is only loaded into releasable SVs regenerated after endocytosis whereas SypHTm labels all SVs including the non-releasable SVs, which certainly decreased the relative fluorescence change^47^. These notions are supported by the small pHTm fluorescence increase upon high K^+^ stimulation in the control. Furthermore, it is possible that there was a slow but consistent retrieval of surfaced SV proteins to endosomes or lysosomes, which could be accelerated by stimulation-induced compensatory endocytosis. In summary, all three concentrations of NH_4_Cl profoundly alter SV behavior, which cannot be completely recovered by the subsequent 5-minute washout.

## Discussion

Collectively, our electrophysiological and imaging results demonstrate that the extracellular application of NH_4_Cl has a plethora of neuronal effects that are not as transient or reversible as previously assumed. At a relatively low concentration (e.g. 5 mM), NH_4_Cl induces Ca^2+^ release from internal stores, changes in cell membrane conductance of Ca^2+^, and significantly reduces SV release even after a thorough washout. Such long-lasting effects become more profound at higher concentration. At 50 mM we tested, NH_4_Cl depolarizes the neuronal membrane and activates VGCCs to cause a large Ca^2+^ influx. Our findings raise several questions regarding NH_4_Cl usage, intracellular pH manipulation and long-term synaptic change caused by ammonia/ammonium.

Indisputably, genetically encoded pH-FPs provide a versatile tool to study the pH-gradient in designated subcellular structures like endosomes and to analyze membrane protein trafficking associated with both endo- and exocytosis^3–5^. The immediate beneficiaries are neuroscientists studying SVs. The pursuit of high sensitivity led to the invention of pH-FPs like pHuji^6^ and super-ecliptic pHluorin^7^ with nearly complete quenching of their fluorescence at luminal pH. To achieve more exclusive targeting, pH-FPs are often fused to organelle specific proteins like Synaptophysin for synaptic vesicles^23^. While these two improvements enhance the signal-to-noise ratio, they make it much harder to locate pH-FP-expressing synapses and to set up the imaging conditions like focal plane and exposure time without the help of high concentration NH_4_Cl (Fig. S3). Furthermore, measuring the total pH-FPs requires complete neutralization of all intracellular compartments with high concentration NH_4_Cl^22^. Under the assumption that the effects of NH_4_Cl, if there are any, are transient and reversible, repeated imaging of the same cell samples that have already experienced NH_4_Cl is acceptable^22^. Contrary to that assumption, our results call for caution in NH_4_Cl usage, especially prior to image acquisition. Our tests demonstrate that lowering the concentration of NH_4_Cl will not ease that concern, as 5 mM NH_4_Cl still significantly reduces SV release despite 5-minute washout (Figs 3C and 4C). Noticeably, 5 mM NH_4_Cl only partially neutralizes intracellular membrane compartments (Figs 2D and 4B) and hence is unsuitable for measuring total pH-FPs. While reducing the duration of 50 mM NH_4_Cl application to 1 minute may help to mitigate certain effects like E_m_ depolarization (Fig. 1A) and Ca^2+^-influx (Fig. 2B), it is unlikely to prevent increase in [Ca^2+^]_i_ (Fig. 2C1) or spontaneous SV release (Fig. 3B).

Based on our observations, a few measures can be taken to circumvent these concerns. The first is to use pH-FPs with a higher pK_a_ like pHTomato^48^ (Fig. 4) or ratiometric pH-FPs like ratiometric pHluorin^2^ or pHluorin2^50^ so that basal fluorescence can be readily detected without the use of NH_4_Cl. The cost of this solution is a reduced fluorescence change and thus less detection capability. Second, expressing cells or organelles can be co-labeled by another pH-insensitive protein with separable fluorescence emission like pHluorin-mKate2^51^, which also has drawbacks like fluorescence bleed-through or fewer options for other fluorescent labels. Third, if high concentration NH_4_Cl has to be applied, likely for all quantitative analyses, it should be done only at the end of imaging^22^ and the NH_4_Cl-exposed cells should not be reused.

Our tests simulating the pre-treatment of NH_4_Cl also provide some insights into the discrepancies between studies using pH-FPs and other methods like Styryl dyes or amperometry. A few pHluorin-based studies concluded that clathrin-mediated endocytosis was the predominant route for SV retrieval after exocytosis^23, 25^, whereas imaging-based studies relying on GFP quenching^52^, FM dyes^53^ or photoluminescent nanoparticles^54^ and electrophysiology-based studies using amperometry and capacitance^55, 56^ suggested that a transient and reversible mode of SV exo-/endocytosis (a.k.a. kiss-and-run, K&R) frequently occurred. We reason that the application of NH_4_Cl prior to the imaging of SV-specific pH-FPs (i.e. Synaptophysin-pHluorin) in the former might decrease the pool of SVs that favor K&R^54^ or alter SV fusion modes by altering presynaptic [Ca^2+^]_i_^57–59^. Future tests combining pH-FPs with other detection methods will be useful to address that. Another interesting study of single SV behavior using SynaptopHluorin^49^ provided some support for this notion. In particular, a considerable amount of SV endocytosis without the proceeding exocytosis was observed upon stimulation, very much resembling the phenomenon we observed after cells were pretreated with NH_4_Cl (Figs 3C and 4C).

It is surprising and certainly against the conventional understanding that the application of NH_4_Cl, even as low as 5 mM, actually results in a long-lasting change in SV release (Figs 3C and 4C) regardless of the relative short duration (5 minutes) and extensive washout. By electrophysiological and imaging measurements, both the E_m_ and the intracellular pH returned to normal after a 5-minute washout (Figs 1 and 2D). Hence the SV changes cannot be directly caused by changes in E_m_ or intracellular pH. On the other hand, Ca^2+^-imaging did hint that synaptic [Ca^2+^]_i_ or internal Ca^2+^-stores may be associated with long-lasting SV alteration since the increase of Ca^2+^ indicator fluorescence did extend to the washout period, especially in the case of 50 mM NH_4_Cl (Fig. 2B). Since Ca^2+^ regulates numerous cell functions including almost every aspect of synaptic transmission, it is intriguing to us that even 5 mM NH_4_Cl has a long-term impact on internal Ca^2+^ stores and the plasma membrane Ca^2+^ conductance. More importantly, both of them are critical to [Ca^2+^]_i_ homeostasis and reciprocally modulate each other, which clearly helps to multiply the [Ca^2+^]_i_ response to NH_4_Cl and extend its timescale. Particularly of interest, mitochondria, often positioned near presynaptic terminals, are the predominant energy powerhouse supporting SV release^60^ and a major Ca^2+^-store influencing cytosolic Ca^2+^ and the turnover of SVs^61, 62^. Intriguingly, the intermembrane space of mitochondria is acidic because protons are pumped across the inner membrane while electrons flow through the respiratory chain. This pH gradient is also coupled to Ca^2+^ uptake into the mitochondrial matrix. Therefore, NH_4_Cl-induced global neutralization will not only halt mitochondrial respiration but also Ca^2+^ uptake^31^, which can be difficult to recover promptly. Moreover, these mitochondrial changes can influence the metabolism of many important molecules like glutamine, GABA and lactose^63^, and can cause presynaptic energy shortage, which is particularly detrimental to endocytosis^64^. All of these may explain the reduction of SypHTm retrieval we had observed (Fig. 4D).

In addition to its direct action on neurons and synapses, NH_4_Cl can also exert its effect via astrocytes. As revealed by the studies of hyperammonemia, excessive ammonia in the extracellular space can disrupt glutamate-glutamine metabolism in astrocytes, and cause the elevation of extracellular glutamate^65^. Consequently, excessive glutamate acts on glutamate receptors and transporters to induce excitotoxicity^66^. While most of the hyperammonemia pathology occurs at a sub-millimolar concentration in a chronic fashion, the effect of acute but higher concentration of NH_4_Cl may be more destructive than that of hyperammonemia. In summary, our results raise the concern of intracellular pH manipulation by high concentration NH_4_Cl, which exerts multiple neuronal effects. Furthermore, some of the effects are clearly long-lasting. Thorough investigation of the neuronal impact of NH_4_Cl will not only help to clarify the discrepancies between previous studies, but will also help to unravel various factors associated with presynaptic modulations through [Ca^2+^]_i_ and mitochondrial bioenergetics.

## Materials and Methods

### Cell culture and gene cloning

All murine procedures and all experimental protocols and methods were approved by the Vanderbilt University Animal Care and Use Committee (VUACUC) (#M1500052) and were performed in accordance with the VUACUC approved guidelines and regulations. For all experiments, primary cultures of dissociated postnatal rat hippocampal cells were prepared as previously described^67^ with some modifications. Briefly, rat hippocampi (CA1-CA3) were dissected from P0 or P1 Sprague-Dawley rats (both sexes) and dissociated into a single-cell suspension with a 10-minute incubation in Trypsin-EDTA (Life Technologies) followed by gentle trituration using three glass pipettes of different diameters (~1 mm, 0.5 mm, and 0.2 mm), sequentially. Dissociated cells were recovered by centrifugation (×200 g, 5 minutes) at 4 °C and re-suspended in plating media composed of Minimal Essential Medium (MEM, Life Technologies) with (in mM) 27 glucose, 2.4 NaHCO_3_, 0.00125 transferrin, 2 L-glutamine, 0.0043 insulin and 10%/vol fetal bovine serum (FBS, Omega). 100 μL of cell suspension was added onto round 12 mm-∅ glass coverslips (200–300 cells/mm^2^) pre-coated with Matrigel (Life Technologies) placed in 24-well plates (ThermoScientific). Cells were allowed to adhere to the coverslip surfaces for 30–60 minutes before the addition of 1 mL plating media. After 1–2 days in culture, additional 1 mL media containing (in mM) 27 glucose, 2.4 NaHCO_3_, 0.00125 transferrin, 0.5 L-glutamine, 2 Ara-C, 1%/vol B27 supplement (Life Technologies) and 5%/vol FBS was added. Ara-C in the culture media efficiently prevented astroglia proliferation. Experiments were performed between DIV 12 and 18 (when synaptic transmission was well established).

The Synaptophysin-pHTomato plasmid (pTGW-UAS-SypHTm) was a gift from Dr. Yulong Li (Peking University, China). The SypHTm fragment was cloned into a mammalian expression vector (pCDNA3.1) containing human synapsin1 promoter by Gibson Assembly as described previously^68^. The DNA primers for the Gibson Assembly were 5′-CGTGCCTGAGAGCGCAGTCGAATTAGCTTGGTACCATGGACGTGGTGAATCAGCTGGTGG-3′ (forward primer) and 5′-TAGAATAGGGCCCTCTAGATGCATGCTCGAGCGGCCGCTTACATCTGATTGGAGAAGGAG-3′ (reverse primer). The resulting plasmid was verified by DNA sequencing.

### Electrophysiology

Whole-cell current clamp recordings were performed on neurons from 12–18 DIV cultures using a Multi-Clamp 700B amplifier, digitized through a Digidata 1440 A, and interfaced via pCLAMP 10 software (all from Molecular Devices). All recordings were performed at room temperature. E_m_ in individual neurons was recorded in the presence of a blocker cocktail: 1-µM tetrodotoxin (TTX), 10-µM 2,3-dihydroxy-6-nitro-7-sulfamoylbenzo[f]quinoxaline-2,3-dione (NBQX, Abcam), 20-µM D-(-)-2-Amino-5-phosphonopentanoic acid (D-AP5, Abcam), and 10-µM Bicuculline (Abcam). Patch pipettes were pulled from borosilicate glass capillaries with resistances ranging from 3–6 MΩ when filled with pipette solution. The bath solution (Tyrode’s saline) contained (in mM): 150 NaCl, 4 KCl, 2 MgCl_2_, 2 CaCl_2_, 10 N-2 hydroxyethyl piperazine-n-2 ethanesulphonic acid (HEPES), 10 glucose, pH 7.35. In 5 mM/10 mM/50 mM NH4Cl-containing solutions NaCl was substituted equimolarly. The pipette solution contained (in mM): 130 Potassium Gluconate, 7 KCl, 2 NaCl, 1 MgCl_2_, 10 HEPES, 0.4 ethylene glycol-bis-(aminoethyl ethane)-N,N,N’,N’-tetraacetic acid (EGTA), 2 MgATP, 0.3 GTP-Tris, pH 7.2. All signals were digitized at 20 kHz, filtered at 2 kHz, and analyzed offline with Clampfit software (Molecular Devices). Input resistance (R_in_) was calculated as the ratio of V/I. Voltage was measured in response to 1-second -10 pA pulses in current clamp mode. All data were exported to and processed in Microsoft Excel.

### Live cell fluorescence imaging and analysis

All live cell imaging was performed on a Nikon Eclipse Ti inverted microscope with a 20x Plan Apo CV objective (N.A. 0.75) and aided by 1.5x optical lens in front of the camera. Cells cultured on 12 mm coverslips were mounted in an RC-26G imaging chamber (Warner Instruments) bottom-sealed with a 24 × 40 mm size 0 cover glass (Fisher Scientific). The chamber was fixed in a PH-1 platform (Warner Instruments) placed on the microscope stage. Solution exchange was achieved via gravity perfusion controlled by a VC-6 valve control system and a 6-channel manifold (Warner Instruments) with a constant rate of ~50 μL/sec which allowed a complete change of bath solution in the recording chamber within 30 s. Image acquisition and synchronized perfusion were controlled via Micro-manager software. For every fluorophore, the acquisition settings including excitation power, fluorescence filter set (excitation, dichroic and emission filters), exposure time, camera gain and frame rate were all kept the same among different samples.

### FM1-43 destaining

Glutamate receptor blockers (with 10 μM NBQX and 20 μM D-AP5) were present throughout the imaging experiments. Before sequential NH_4_Cl application, synaptically mature primary rat hippocampal neurons (DIV 12–18) were incubated with 10 µM FM1-43 (i.e., SynaptoGreen C4, Biotium) for 0.5–1 hour at 37 °C in 5% CO_2_ incubator to ensure loading of the dye into synaptic vesicles through spontaneous endocytosis. For simulating NH_4_Cl pretreatment and washout, 10 µM FM1-43 in Tyrode’s solution with 90 mM K^+^ was applied to the cells on coverslips before 5-minute washout of cell surface FM1-43 and before NH_4_Cl pretreatments. FM1-43 imaging was done using a fluorescence filter set: Ex. 460/50; DIC: 495LP; Em: 510/25BP. All optical filters and dichroic mirrors were purchased from Chroma or Semrock. Images were taken at 0.1 Hz rate with the same acquisition settings (excitation light intensity, exposure time and EM gain) among different samples. Image analysis was done in ImageJ. Regions of interest (ROIs) were selected by using the same fluorescence intensity threshold across different samples. Average intensity of every ROI and average background intensities from four cell-free regions in every image stack were exported to Excel. The FM1-43 signal in every ROIs were calculated as F/F_0_, in which F_0_ is the average of the first ten frames and both F and F_0_ are background subtracted.

### SypHTm and Calcium imaging

Neurons were transiently transfected with SypHTm construct at DIV 7 and imaged at DIV 12–18. Glutamate receptor blockers (NBQX and DAP-5) were present throughout the imaging experiments. Fluorescence signal was visible in normal Tyrode’s bath condition with the following filter set: Ex. 560/40, DIC 585LP, Em. 610/20. Changes of SypHTm fluorescence that reflect de-acidification of synaptic vesicles were monitored in the presence of 5 mM, 10 mM and 50 mM NH_4_Cl. In parallel, effects of NH_4_Cl on [Ca^2+^]_i_ were monitored by imaging Fluo-4 fluorescence. For that, SypHTm-transfected cultures were incubated with Fluo-4, AM Ester (1 μM, ThermoFisher Scientific) for 30–60 min at 37 °C in 5% CO_2_ before washout. For SypHTm or Fluo-4, images were taken at 0.2 Hz rate with the same acquisition settings (excitation light intensity, exposure time and EM gain) among different samples. In case of simulating NH_4_Cl pretreatment with washout, cultures transfected with SypHTm were used without preloading of Fluo-4-AM. Image analysis was done in ImageJ. ROIs were selected by using the same fluorescence intensity (Fluo-4 for somatodendritic areas and SypHTm for synaptic areas) threshold across different samples. Average intensity from every ROI and average background intensities from four cell-free regions in every image stack was exported to Excel. The fluorescence signal in every ROI was calculated as F/F_0_, in which F_0_ is the average of the first ten frames and both are background subtracted. And in case of simulating NH_4_Cl pretreatment with washout, the fluorescence signal in every ROI was calculated as F/F_max_, in which F_max_ is the difference between the maximal SypHTm intensities in every ROI during final NH4Cl perfusion and the minimal SypHTm intensities during final pH5.5 perfusion (both are background subtracted).

### BECEF imaging

BCECF, AM Ester (Biotium, 1 μM) was added to cell culture medium for 20 min at 37 °C in 5% CO_2_. Fluorescent signal was detected using two filter sets: Ex. 405/20, DIC 495LP, Em. 510/25 and Ex. 475/20, DIC 490LP, Em. 510/25. Images were collected for 21 minutes at 0.2 Hz rate using the solution perfusion protocol with the following order: 1 min baseline in Tyrode’s, 5 min in NH4Cl (5 mM), 5 min NH4Cl (10 mM), 5 min NH4Cl (5 mM), 5 min wash in the normal Tyrode’s solution. Glutamate receptor blockers (NBQX and DAP-5) were present throughout the imaging experiments. ROIs were selected by using the same fluorescence intensity threshold in the Ex = 475/20 nm channel across different samples. Average intensity of all ROIs and average background intensities of four cell-free regions in every image stack was exported to Excel. The two channels of fluorescence signal in every ROI were registered and used to calculate the ratio (R = F_480_/F_440_, both F_480_ and F_440_ are background subtracted), and R_0_ is the average of the first ten frames.

### Data Analysis

To determine the minimum number of ROIs for FM1-43 destaining, a power analysis was performed using G*Power^69^. An effect size of 25% was estimated with the error probability set to 0.05, power to 0.95 and an expected standard deviation of 40% was chosen based on FM destaining experiments performed in the lab. A sample size of 53 is needed to achieve significance with a two-tailed Student’s *t*-test. Three separate trials per condition with 79 ROIs each, for a total n of 237, was judged to be more than sufficient. To detect an effect size of 10% with error probability 0.05 and power 0.8 for the SypHTm baseline data in Fig. 4, 105 total ROIs from 3 trials was deemed sufficient. SypHTm ROIs were excluded if NH4Cl and pH 5.5 application did not achieve a raw fluorescence value higher or lower, respectively, than the baseline values. All image processing was performed in ImageJ as described previously^70^. All experiments were performed in two to three different batches of cell cultures. All values presented are mean ± s.e.m. For calculating statistical significance, the Student’s *t*-test was used for 2-group comparison, and one-way analysis of variance (ANOVA) followed by the Tukey-Kramer method as post-hoc analysis was used for comparing three or more groups.

### Data Availability Statement

The full data set supporting this paper is available from the corresponding author upon request.

## Electronic supplementary material

Supplementary Information

Supplementary Video 1

Supplementary Video 2

Supplementary Video 3
