# Supplementary material for: Ammonium chloride alters neuronal excitability and synaptic vesicle release
Source: Sci Rep. 2017 Jul 11;7:5061. doi: 10.1038/s41598-017-05338-5 (PMC5505971; doi:10.1038/s41598-017-05338-5)
Supplement: Supplementary file 1 — Supplementary Information [file 41598_2017_5338_MOESM1_ESM.pdf]

Supplementary Online Materials:

Title Page

Title

Ammonium chloride alters neuronal excitability and synaptic vesicle release

Authors

Roman M. Lazarenko[1], Claire E. DelBove[1], Claire E. Strothman[1], Qi Zhang[1*]

[1] Department of Pharmacology, Vanderbilt University, 23rd Avenue South at Pierce Street, Nashville, TN 37232 USA

* Corresponding author:

Room 410A, Robinson Research Bldg

Dept. Pharmacology, Vanderbilt University Medical Center

23rd Ave. S. at Pierce Ave.

Nashville, TN 37232-6600

Phone: (615)-875-7620

E-mail: qi.zhang@vanderbilt.edu

1. Supplementary videos

1) Intracellular calcium changes in response to the consecutive application of normal Tyrode's solution, 5, 10 and 50 mM NH_4Cl and washout. 12-DIV rat hippocampal culture was loaded with Fluo-4, AM for 30 minutes before mounting on an imaging chamber. External solutions were applied via gravity perfusion and the switching of bath solutions was automatic and synchronized to image acquisition.

2) Synaptic vesicles release in response to the consecutive application of normal Tyrode's solution, 5, 10 and 50 mM NH_4Cl and washout. 12-DIV rat hippocampal culture was loaded with FM1-43 (5 μM) by 90 mM K^+ for 2 minutes in the imaging chamber. External solutions were applied via gravity perfusion and the switching of bath solutions was automatic and synchronized to image acquisition.

3) The fluorescence of Synaptophysin-pHTomato changes in response to the consecutive application of normal Tyrode's solution, 5, 10 and 50 mM NH_4Cl and pH5.5 Tyrode's solution. Rat hippocampal culture was transfected on 10 DIV and imaged on 12 DIV. External solutions were applied via gravity perfusion and the switching of bath solutions was automatic and synchronized to image acquisition.

2. Supplementary figure

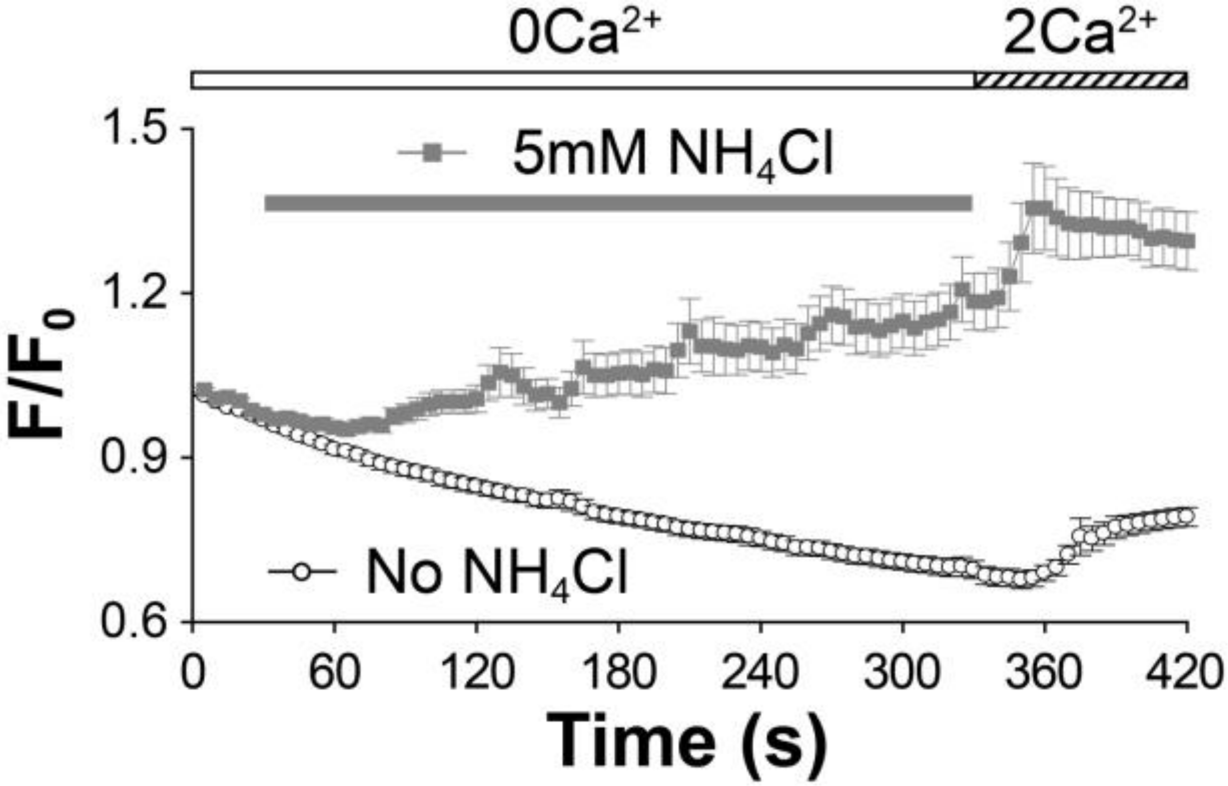

Figure S1. $[Ca^{2+}]_i$ changes in response to extracellular Ca^{2+} change and NH_4Cl. When extracellular Ca^{2+} was changed from 0 to 2 mM, a small and gradual $[Ca^{2+}]_i$ increase occurred (white circles). In comparison, 5 mM NH4Cl without Ca^{2+} also induced a slow $[Ca^{2+}]_i$ increase and the switch to 2 mM extracellular Ca2+ afterwards quickly induced additional $[Ca^{2+}]_i$ increase (gray squares). For each group, 10 ROIs/FOV were randomly selected and 4 FOVs were analyzed for each condition.

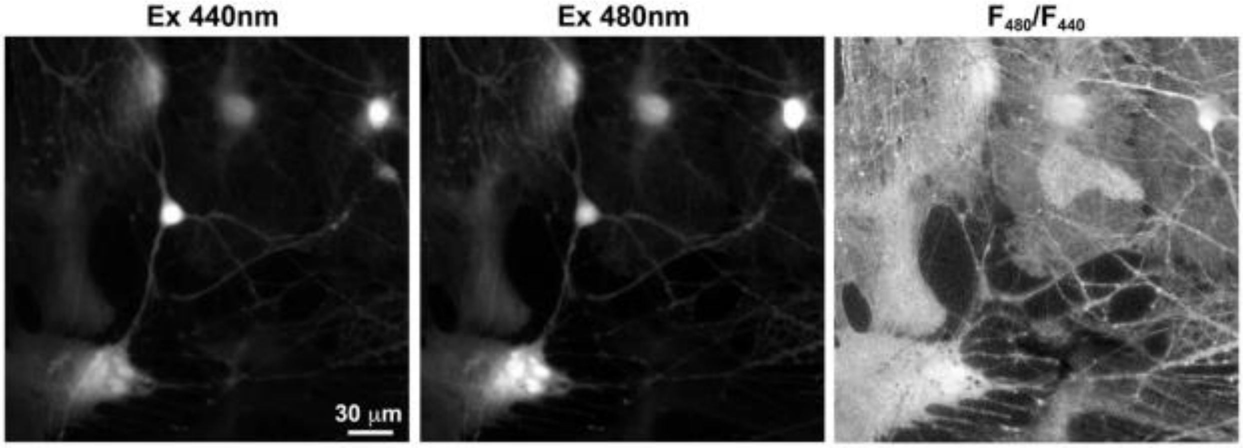

Figure S2. BCECF for measuring intracellular pH. Sample images of neuronal culture loaded with BCECF-AM and imaged with 440 nm (left image) or 480 nm (middle image) excitation. The F_{480}/F_{440} ratio (right image) reports intracellular pH.

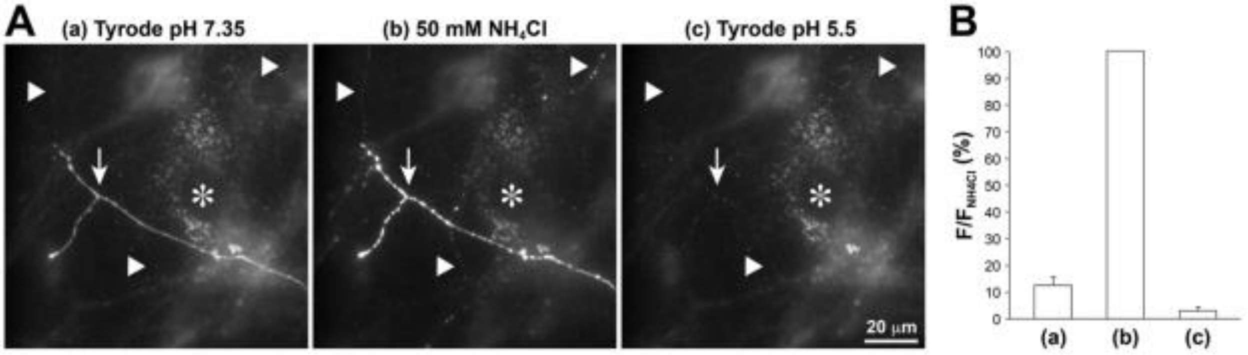

Figure S3. NH4Cl is necessary to visualize Synaptophysin-pHluorin-positive synapses. (**A**) Sample images of rat hippocampal culture transfected with Synaptophysin-pHluorin (SypH). In this case, we used a neurite with abnormally high green fluorescence (indicated by an arrow) to determine the focal plane and acquisition settings. Generally, neurons with such high SypH expression are morphologically abnormal (e.g. swollen) and unresponsive to depolarization, although they may still respond to 50 mM NH_4Cl. In the normal Tyrode's solution (pH 7.35), three neurites with a normal level of SypH expression (indicated by arrowheads) are nearly invisible (left image). After the application of 50 mM NH_4Cl, those three neurites and the fluorescent puncta within become detectable and so do the puncta in the bright neurites (middle image). Finally, the perfusion of pH 5.5 Tyrode's solution quenches almost all green fluorescence but not auto-fluorescence from astrocytes (indicated by asterisk) (right image). Scale bar, 20 μm. (**B**) Average green fluorescence in neurites in the three solutions. The intensity values in every region of interest is normalized to the maximum intensity value obtained in 50 mM NH_4Cl (i.e. F/F_{NH4Cl} %, and thus all values for **b** are 1 without variance). For every condition, 30 randomly chosen regions of interest (i.e. all n = 30 ROIs) were used for analysis. (a, b and c) represents three conditions annotated in (**A**).
